# Supplementary material for: Pyruvate Dehydrogenase Kinase 1 inhibition mediated oxidative phosphorylation enhancement in cartilage promotes osteoarthritis progression
Source: BMC Musculoskelet Disord. 2023 Jul 20;24:597. doi: 10.1186/s12891-023-06585-6 (PMC10357736; doi:10.1186/s12891-023-06585-6)
Supplement: Supplementary file 1 — Additional file 1. [file 12891_2023_6585_MOESM1_ESM.zip › KEGG.Gsea.1653622432464/KEGG_CYSTEINE_AND_METHIONINE_METABOLISM.html]

Details for gene set KEGG\_CYSTEINE\_AND\_METHIONINE\_METABOLISM[GSEA]

|  || Dataset | 55457\_collapsed\_to\_symbols.cls.cls #OA\_versus\_normal.cls.cls #OA\_versus\_normal\_repos |
| Phenotype | cls.cls#OA\_versus\_normal\_repos |
| Upregulated in class | OA |
| GeneSet | KEGG\_CYSTEINE\_AND\_METHIONINE\_METABOLISM |
| Enrichment Score (ES) | 0.28576446 |
| Normalized Enrichment Score (NES) | 1.0384963 |
| Nominal p-value | 0.40238094 |
| FDR q-value | 0.5431091 |
| FWER p-Value | 1.0 |
Table: GSEA Results Summary

  

Fig 1: Enrichment plot: KEGG\_CYSTEINE\_AND\_METHIONINE\_METABOLISM      
 Profile of the Running ES Score & Positions of GeneSet Members on the Rank Ordered List

  

| SYMBOL | TITLE | RANK IN GENE LIST | RANK METRIC SCORE | RUNNING ES | CORE ENRICHMENT || 1 | GOT1 | glutamic-oxaloacetic transaminase 1 [Source:HGNC Symbol;Acc:HGNC:4432] | 59 | 0.974 | 0.0966 | Yes |
| 2 | DNMT3A | DNA methyltransferase 3 alpha [Source:HGNC Symbol;Acc:HGNC:2978] | 406 | 0.704 | 0.1429 | Yes |
| 3 | APIP | APAF1 interacting protein [Source:HGNC Symbol;Acc:HGNC:17581] | 643 | 0.607 | 0.1877 | Yes |
| 4 | MAT2B | methionine adenosyltransferase 2B [Source:HGNC Symbol;Acc:HGNC:6905] | 715 | 0.589 | 0.2434 | Yes |
| 5 | GOT2 | glutamic-oxaloacetic transaminase 2 [Source:HGNC Symbol;Acc:HGNC:4433] | 1308 | 0.457 | 0.2449 | Yes |
| 6 | TAT | tyrosine aminotransferase [Source:HGNC Symbol;Acc:HGNC:11573] | 1979 | 0.363 | 0.2305 | Yes |
| 7 | ADI1 | acireductone dioxygenase 1 [Source:HGNC Symbol;Acc:HGNC:30576] | 2004 | 0.360 | 0.2661 | Yes |
| 8 | CDO1 | cysteine dioxygenase type 1 [Source:HGNC Symbol;Acc:HGNC:1795] | 2635 | 0.287 | 0.2469 | Yes |
| 9 | DNMT1 | DNA methyltransferase 1 [Source:HGNC Symbol;Acc:HGNC:2976] | 2836 | 0.267 | 0.2591 | Yes |
| 10 | AHCYL2 | adenosylhomocysteinase like 2 [Source:HGNC Symbol;Acc:HGNC:22204] | 3099 | 0.240 | 0.2637 | Yes |
| 11 | MTAP | methylthioadenosine phosphorylase [Source:HGNC Symbol;Acc:HGNC:7413] | 3324 | 0.219 | 0.2690 | Yes |
| 12 | DNMT3L | DNA methyltransferase 3 like [Source:HGNC Symbol;Acc:HGNC:2980] | 3393 | 0.212 | 0.2858 | Yes |
| 13 | SDS | serine dehydratase [Source:HGNC Symbol;Acc:HGNC:10691] | 4579 | 0.106 | 0.2047 | No |
| 14 | LDHAL6B | lactate dehydrogenase A like 6B [Source:HGNC Symbol;Acc:HGNC:21481] | 5040 | 0.070 | 0.1762 | No |
| 15 | CBS | cystathionine beta-synthase [Source:HGNC Symbol;Acc:HGNC:1550] | 5285 | 0.049 | 0.1623 | No |
| 16 | AHCY | adenosylhomocysteinase [Source:HGNC Symbol;Acc:HGNC:343] | 5307 | 0.047 | 0.1656 | No |
| 17 | ENOPH1 | enolase-phosphatase 1 [Source:HGNC Symbol;Acc:HGNC:24599] | 5615 | 0.023 | 0.1441 | No |
| 18 | AHCYL1 | adenosylhomocysteinase like 1 [Source:HGNC Symbol;Acc:HGNC:344] | 5837 | 0.006 | 0.1275 | No |
| 19 | TRDMT1 | tRNA aspartic acid methyltransferase 1 [Source:HGNC Symbol;Acc:HGNC:2977] | 6204 | -0.021 | 0.1013 | No |
| 20 | BHMT | betaine--homocysteine S-methyltransferase [Source:HGNC Symbol;Acc:HGNC:1047] | 7250 | -0.099 | 0.0304 | No |
| 21 | LDHC | lactate dehydrogenase C [Source:HGNC Symbol;Acc:HGNC:6544] | 7603 | -0.127 | 0.0163 | No |
| 22 | LDHB | lactate dehydrogenase B [Source:HGNC Symbol;Acc:HGNC:6541] | 7718 | -0.135 | 0.0214 | No |
| 23 | SRM | spermidine synthase [Source:HGNC Symbol;Acc:HGNC:11296] | 7735 | -0.136 | 0.0344 | No |
| 24 | MPST | mercaptopyruvate sulfurtransferase [Source:HGNC Symbol;Acc:HGNC:7223] | 8457 | -0.190 | -0.0019 | No |
| 25 | MAT1A | methionine adenosyltransferase 1A [Source:HGNC Symbol;Acc:HGNC:6903] | 8753 | -0.213 | -0.0027 | No |
| 26 | SMS | spermine synthase [Source:HGNC Symbol;Acc:HGNC:11123] | 8884 | -0.223 | 0.0104 | No |
| 27 | MTR | 5-methyltetrahydrofolate-homocysteine methyltransferase [Source:HGNC Symbol;Acc:HGNC:7468] | 9213 | -0.249 | 0.0107 | No |
| 28 | DNMT3B | DNA methyltransferase 3 beta [Source:HGNC Symbol;Acc:HGNC:2979] | 10089 | -0.330 | -0.0229 | No |
| 29 | AMD1 | adenosylmethionine decarboxylase 1 [Source:HGNC Symbol;Acc:HGNC:457] | 10415 | -0.363 | -0.0105 | No |
| 30 | CTH | cystathionine gamma-lyase [Source:HGNC Symbol;Acc:HGNC:2501] | 11108 | -0.444 | -0.0181 | No |
| 31 | LDHA | lactate dehydrogenase A [Source:HGNC Symbol;Acc:HGNC:6535] | 11134 | -0.448 | 0.0264 | No |
| 32 | MAT2A | methionine adenosyltransferase 2A [Source:HGNC Symbol;Acc:HGNC:6904] | 12852 | -1.066 | 0.0037 | No |
Table: GSEA details [plain text format]

  

Fig 2: KEGG\_CYSTEINE\_AND\_METHIONINE\_METABOLISM      
 Blue-Pink O' Gram in the Space of the Analyzed GeneSet

  

Fig 3: KEGG\_CYSTEINE\_AND\_METHIONINE\_METABOLISM: Random ES distribution      
 Gene set null distribution of ES for **KEGG\_CYSTEINE\_AND\_METHIONINE\_METABOLISM**
